# Reticular Formation Connections Underlying Horizontal Gaze: The Central Mesencephalic Reticular Formation (cMRF) as a Conduit for the Collicular Saccade Signal

**DOI:** 10.3389/fnana.2017.00036

**Published:** 2017-04-25

**Authors:** Niping Wang, Eddie Perkins, Lan Zhou, Susan Warren, Paul J. May

**Affiliations:** ^1^Department of Neurobiology and Anatomical Sciences, University of Mississippi Medical CenterJackson, MS, USA; ^2^Department of Periodontics and Preventive Sciences, University of Mississippi Medical CenterJackson, MS, USA; ^3^Department of Neurosurgery, University of Mississippi Medical CenterJackson, MS, USA; ^4^Department of Internal Medicine, G.V. Montgomery Veterans Administration Medical CenterJackson, MS, USA; ^5^Department of Neurology, University of Mississippi Medical CenterJackson, MS, USA; ^6^Department of Ophthalmology, University of Mississippi Medical CenterJackson, MS, USA

**Keywords:** oculomotor, eye movement, saccade, superior colliculus, PPRF, gaze

## Abstract

The central mesencephalic reticular formation (cMRF) occupies much of the core of the midbrain tegmentum. Physiological studies indicate that it is involved in controlling gaze changes, particularly horizontal saccades. Anatomically, it receives input from the ipsilateral superior colliculus (SC) and it has downstream projections to the brainstem, including the horizontal gaze center located in the paramedian pontine reticular formation (PPRF). Consequently, it has been hypothesized that the cMRF plays a role in the spatiotemporal transformation needed to convert spatially coded collicular saccade signals into the temporally coded signals utilized by the premotor neurons of the horizontal gaze center. In this study, we used neuroanatomical tracers to examine the patterns of connectivity of the cMRF in macaque monkeys in order to determine whether the circuit organization supports this hypothesis. Since stimulation of the cMRF produces contraversive horizontal saccades and stimulation of the horizontal gaze center produces ipsiversive saccades, this would require an excitatory cMRF projection to the contralateral PPRF. Injections of anterograde tracers into the cMRF did produce labeled terminals within the PPRF. However, the terminations were denser ipsilaterally. Since the PPRF located contralateral to the movement direction is generally considered to be silent during a horizontal saccade, we then tested the hypothesis that this ipsilateral reticuloreticular pathway might be inhibitory. The ultrastructure of ipsilateral terminals was heterogeneous, with some displaying more extensive postsynaptic densities than others. Postembedding immunohistochemistry for gamma-aminobutyric acid (GABA) indicated that only a portion (35%) of these cMRF terminals are GABAergic. Dual tracer experiments were undertaken to determine whether the SC provides input to cMRF reticuloreticular neurons projecting to the ipsilateral pons. Retrogradely labeled reticuloreticular neurons were predominantly distributed in the ipsilateral cMRF. Anterogradely labeled tectal terminals were observed in close association with a portion of these retrogradely labeled reticuloreticular neurons. Taken together, these results suggest that the SC does have connections with reticuloreticular neurons in the cMRF. However, the predominantly excitatory nature of the ipsilateral reticuloreticular projection argues against the hypothesis that this cMRF pathway is solely responsible for producing a spatiotemporal transformation of the collicular saccade signal.

## Introduction

Humans continuously examine their environment through a series of gaze changes involving saccadic eye movements and, in some cases, accompanying head movements that require accurate and precise coordination. Consequently, when neurological disorders or deficits interfere with gaze, the outcome can be debilitating (Leigh and Zee, 2015). Numerous physiological studies have demonstrated that the superior colliculus (SC) plays an important role in selecting gaze targets (Wurtz and Goldberg, 1972; Munoz and Guitton, 1986; Paré et al., 1994; Freedman and Sparks, 1997; Sparks et al., 2001). The connections of the SC with pontine gaze centers are by way of the crossed tectobulbospinal tract or predorsal bundle, which targets the contralateral paramedian pontine reticular formation (PPRF; Harting, 1977; May, 2006; Basso and May, 2017). The PPRF contains premotor neurons that initiate horizontal saccades through their inputs to motoneurons and internuclear neurons found in the ipsilateral abducens nucleus (Hepp et al., 1989; Horn, 2006). The PPRF has been hypothesized to generate a command for horizontal saccades by extracting it from saccadic signals provided by the SC (Fuchs et al., 1985; Moschovakis et al., 1996). It contains premotor neurons that confer a code for the size and speed of the movement upon the abducens nucleus (Luschei and Fuchs, 1972; Keller, 1979; Horn, 2006). These premotor cells are referred to as medium lead saccadic burst neurons due to their high rate of firing in conjunction with saccades and the fact they begin firing before the short lead burst of the motoneurons (Fuchs et al., 1985; Moschovakis et al., 1996). They specifically fire for ipsiversive saccades and are silent for contraversive ones. This role for the PPRF is supported by stimulation studies and evidence that lesions in this region produced horizontal gaze palsies (Goebel et al., 1971; Cohen and Komatsuzaki, 1972; Kato et al., 2006).

The pontine portion of the horizontal gaze center consists of the medial region of the nucleus reticularis pontis oralis (NRPO) and nucleus reticularis pontis caudalis (NRPC), which houses medium lead excitatory burst neurons (EBNs). The EBNs activate the ipsilateral abducens motoneurons causing the ipsilateral eye to abduct (for review Henn and Cohen, 1976; Hepp and Henn, 1983; Strassman et al., 1986a; Hepp et al., 1989). They also contact internuclear neurons in the abducens nucleus that activate medial rectus motoneurons in the contralateral oculomotor nucleus, so that a comparable movement is made by the opposite eye, producing conjugate eye movements. The horizontal gaze center also includes the medial region of the nucleus paragigantocellularis dorsalis of the rostral medulla, as it houses inhibitory burst neurons (IBNs). IBN firing is similar in timing and intensity to that of EBNs during saccades and fixation (Hikosaka and Kawakami, 1977; Hikosaka et al., 1978; Yoshida et al., 1982). IBNs suppress the activity of antagonist muscles (the contralateral lateral rectus and ipsilateral medial rectus) through a glycinergic, crossed inhibitory projection to the motoneurons and internuclear neurons in the abducens nucleus (Yoshida et al., 1982; Strassman et al., 1986b).

While this circuitry is well worked out, the precise pathway(s) whereby the SC, which chooses saccade targets, sends this information to the EBNs and IBNs is still a matter of argument. Furthermore, the manner in which collicular signals are converted into the necessary burst neuron firing patterns is still obscure (see Moschovakis et al., 1998). For example, there is conflicting evidence with respect to whether the SC directly targets EBNs and IBNs. Raybourn and Keller (1977) could not find action potentials in monkey EBNs whose latencies were short enough following electrical stimulation of the SC to suggest monosynaptic input. Evidence from cats suggests monosynaptic tectal projections are supplied to PPRF premotor neurons (Grantyn et al., 1980, 1987; Grantyn and Berthoz, 1987; Izawa et al., 1999), and more specifically to IBNs (Hikosaka and Kawakami, 1977; Grantyn et al., 1979; Takahashi et al., 2005). Furthermore, Chimoto et al. (1996) found evidence for monosynaptic tectal inputs to cat EBNs, when the omnipause inhibition was gated. However, Keller was not able to reproduce this effect in monkeys (Keller et al., 2000). There is evidence that long lead burst neurons (LLBNs) in the rostral brainstem receive direct inputs from the SC (Luschei and Fuchs, 1972; Hepp and Henn, 1983; Scudder et al., 1996a), and send efferents to medium lead burst neurons. Therefore, it has been suggested that these cells may serve as interneurons between the SC and premotor neurons in primates (Scudder et al., 1996b).

The central mesencephalic reticular formation (cMRF) is one of the structures that contains LLBNs (Waitzman et al., 1996; Handel and Glimcher, 1997). While earlier reports of a saccade-related area within the midbrain reticular formation (MRF) exist (cat: Szentagothai, 1943; Bender and Shanzer, 1964), the cMRF was first described in detail and named by Cohen and Büttner-Ennever (1984) and Cohen et al. (1985). They defined it as an area in the midbrain tegmentum of primates that produces horizontally directed contraversive saccades following electrical stimulation. They suggested three possible roles for the cMRF: saccade triggering, feedback control of saccadic activity and feed forward control of saccade-related activity. A similar area has been identified in goldfish, suggesting this structure is a common vertebrate feature (Angeles Luque et al., 2005; Luque et al., 2006), although its stimulation in fish did not just produce saccades; the animals turned their head and realigned their bodies.

Waitzman et al. (1996) recorded from individual cMRF neurons in awake, behaving monkeys and showed that about three quarters of cMRF neurons discharge before and/or during contraversive, visually guided rapid eye movements, and during contraversive spontaneous saccades in the dark. The number of spikes appeared to correlate with the size of the horizontal component of the saccade. In later reports, they made reversible lesions in this structure (Waitzman et al., 2000a,b). Based on their findings, they subdivided the MRF into a caudal region, the cMRF, where inactivation affects the horizontal component of saccades, and a rostral region, the peri-interstitial nucleus of Cajal portion of the MRF (piMRF), in which inactivation affects the vertical component of saccades. They suggested that a group of LLBNs located in the piMRF play a role in vertical saccadic eye movements, in contrast to the cMRF, whose LLBN activity is more related to the horizontal eye movements.

The targets of the various cell types described by these studies were not antidromically identified. Only the cells that project back upon the SC have been described physiologically (Moschovakis et al., 1988b). The activity of these reticulotectal neurons resembles, in most respects, the activity of the collicular cells providing them input. More recent studies identified separate classes of cMRF neurons in monkey that are associated with saccade metrics, including amplitude, velocity and duration (Cromer and Waitzman, 2006, 2007). These authors proposed that cMRF cells whose firing is most tightly coupled to saccade velocity may represent an intermediate step in the spatiotemporal transformation needed to convert the firing of output cells within the motor map present in the SC into the temporally coded firing of premotor neurons in the PPRF. Thus, these cMRF reticuloreticular cells would receive collicular input and project to the contralateral horizontal gaze center.

There are anatomical reasons why the cMRF is a good candidate for this role. The cMRF receives an extensive, topographically organized input from the SC (Edwards, 1975; Harting, 1977; Cohen and Büttner-Ennever, 1984; Moschovakis et al., 1988a, b; Chen and May, 2000; May et al., 2002). In fact, predorsal bundle axons supply axon collaterals to the cMRF prior to crossing in the dorsal tegmental decussation to supply the PPRF (Grantyn and Grantyn, 1982; Moschovakis et al., 1988b). It has been suggested that the cMRF also has projections to the PPRF (Büttner-Ennever and Büttner, 1988). Specifically, retrograde and anterograde studies in cats indicate the MRF provides a bilateral input to the PRF (Edwards, 1975; Stanton and Greene, 1981).

In light of these findings, we undertook a more detailed investigation of this cMRF projection in a primate, *Macaca fascicularis*, in order to specifically test whether the cMRF has appropriate patterns of connectivity to serve the spatiotemporal transformation of the collicular saccade signal. Biotinylated dextran amine (BDA) or *Phaseolus vulgaris* leucoagglutinin (PhaL) was injected into the cMRF of macaque monkeys, in order to anterogradely labeled reticuloreticular axons. We expected that the crossed projection would be excitatory and the ipsilateral projection would be either inhibitory, or end on inhibitory interneurons, since cMRF stimulation produces contraversive saccades and PPRF stimulation produces ipsiversive saccades, and because cells in both regions display a burst of action potentials when saccades are made in their on direction, but are silent when saccades are made in their off direction. To test whether the ipsilateral pathway was inhibitory, we also prepared material for electron microscopic investigation. Postembedding, gamma-aminobutyric acid (GABA) immunohistochemistry was used to examine the possible GABAergic nature of cMRF reticuloreticular axon terminals and targets in the ipsilateral PPRF. Finally, we tested whether the SC has direct access to this reticuloreticular projection by the use of dual tracer studies. Portions of these results have been presented in abstract form previously (Warren and May, 2004; May et al., 2005; Zhou et al., 2006).

## Materials and Methods

All animal procedures were undertaken in accordance with the animal care and use guidelines of the NIH, including the Guide for the Care and Use of Laboratory Animals, and with the approval of the University of Mississippi Medical Center IACUC. A total of 13 adult or young adult *Macaca fascicularis* monkeys of both sexes underwent surgeries performed with sterile techniques under isoflurane anesthesia (1%–3%; some of these animals were also used in other non-conflicting studies). Animals were sedated with ketamine HCl (10 mg/kg, IM). They were also treated with atropine sulfate (0.2 mg/kg, IV) to reduce airway secretions and dexamethasone (0.4 mg/kg, IV) to minimize cerebral edema. Vital signs, including core temperature and blood O_2_ levels, were monitored and maintained at physiological levels. After the tracers were injected, the aspirated area was filled with hydrated Gelfoam, the incision was closed with suture, and the wound edges were infused with Sensorcaine. Buprenex (0.01 mg/kg, IM) was administered as a postsurgical analgesic.

### Anterograde Tracer Cases

Pressure injections of BDA (Molecular Probes; *n* = 6) were made with a 1.0 μl Hamilton microsyringe attached to a micromanipulator. To avoid the SC, the needle was inserted through the pulvinar (for details, see Wang et al., 2010, 2013). The injection depth was adjusted with respect to the SC surface. Between 0.1 μl and 0.2 μl of a 10.0% solution of BDA was delivered into the left cMRF along each of 1 or 2 penetrations. The same approach was used for the injections of PhaL (*n* = 2). A 2.0% solution in 0.1 M, pH 8.0 phosphate buffered saline solution was injected by means of iontophoresis. A positive current of 7 mA was passed through the PhaL solution, which was held in a glass micropipette with a tip diameter of 20–30 μm. Current was passed for 10–20 min (50% duty cycle, 7 s/pulse).

After a 3 week survival period for BDA injections or a 2 week survival period for PhaL injections, animals were sedated with ketamine HCl (10 mg/kg, IM) and deeply anesthetized with sodium pentobarbital (50 mg/kg, IP). They were perfused via the heart with phosphate buffered saline, followed by a fixative containing 1% paraformaldehyde and 1.25%–1.5% glutaraldehyde in 0.1 M, pH 7.2 phosphate buffer (PB). The brainstem was blocked in the frontal plane, removed and stored in cold 0.1 M, pH 7.2 PB. Frontal sections were cut at 100 μm with a vibratome (Leica VT1000S) for the BDA cases or at 50 μm for the PhaL cases, and collected in PB.

For BDA injections, at least two rostrocaudal 1 in 3 series at 300 μm intervals were reacted to reveal the presence of the tracer. As previously described (Barnerssoi and May, 2016), the sections were incubated overnight at 4°C in a solution containing Avidin D conjugated to horseradish peroxidase (Vector Laboratories, 1:5000) dissolved in 0.05% triton X-100 in 0.1 M, pH 7.2 PB. They were then rinsed with 0.1 M, pH 7.2 PB and reacted in a solution containing 5.0% diaminobenzidine (DAB) dissolved in 0.1 M, pH 7.2 PB. This solution also contained 0.011% hydrogen peroxide, 0.05% nickel ammonium sulfate and 0.05% cobalt chloride. In preparation for light microscopy, the sections were mounted on gelatinized slides, air dried, counterstained with cresyl violet, dehydrated in a graded series of ethanols, cleared in toluene, and cover slipped.

For PhaL injections, at least two rostrocaudal 1 in 3 series at 150 μm intervals were reacted following previously described procedures (Gerfen and Sawchenko, 1984; Perkins et al., 2009). Specifically, the sections were incubated in a 0.3% triton X-100 in 0.1 M, pH 7.2 PB solution for 20 min, rinsed and then placed in a 10.0% solution of normal goat serum in PB, as a blocking agent. Next, they were incubated in a solution containing biotinylated anti-PhaL (0.5% in 0.01 M, pH 7.2 PB solution), first at room temperature for 1 h, and then overnight at 4°C, with agitation. The next day, the sections were incubated in the final solution of an ABC kit (Vector Laboratories) for 1–2 h. After rinsing in a 0.1 M, pH 7.2 PB solution, the HRP tagged Avidin-Biotin complex was revealed as follows. Sections were placed in a 5.0% DAB solution in 0.1 M, pH 7.2 PB for 10 min, and the reaction was then initiated with H_2_O_2_ (0.011%) and allowed to run for up to 30 min. Reacted sections were mounted and prepared for light microscopy in the same manner as the BDA labeled sections.

### Ultrastructural Procedures

Two of these same BDA cases with the most discrete cMRF injections were used for ultrastructural examination. Under a stereomicroscope (Leica Wild M8), small tissue blocks containing labeled terminals were cut out of free-floating sections and collected in 0.1 M, pH 7.2 PB. The details of the EM preparation are provided in previous reports (Wang et al., 2010, 2013). Ultrathin sections for conventional EM analysis were collected on copper mesh grids, while those used for GABA postembedding were collected on nickel slot grids. The latter were processed using rabbit anti-GABA (Sigma) and anti-rabbit IgG conjugated to15 nm gold particles (EM Sciences; for details, see Barnerssoi and May, 2016).

### Dual Tracer Experiments

In dual tracer experiments (*n* = 3), injections of BDA and of wheat germ agglutinin conjugated to horseradish peroxidase (WGA-HRP) were made in sequential surgical procedures. Both tracer injections were placed on the left side. For the first surgery, the injection of the SC was performed following an approach similar to that described for the cMRF injection, above. After cortical aspiration, a 1.0 μl Hamilton injection syringe containing BDA was angled at 30°, tip rostral in the sagittal plane, and visually guided into the colliculus. Injection depth ranges from 1.0 mm to 1.5 mm from the surface. Post injection procedures were the same as for the cMRF injection.

Two to three weeks following the initial surgery, animals had a second surgery to inject a retrograde tracer into the PPRF. Injections into PPRF were done using stereotaxic coordinates for the PPRF (AP = 0.6, ML = 1.5, and DV = 0; Szabo and Cowan, 1984). The initial incision from the first surgery was reopened and the Gelfoam aspirated to reveal the tentorium cerebelli. A small incision was made in the tentorium to allow the injection syringe needle to penetrate the underlying cerebellum. A 1.0 μl Hamilton injection syringe attached to a stereotaxic manipulator set at an angle of 10°, tip rostral in the sagittal plane, was passed through the dorsal surface of the cerebellum to penetrate the PPRF. Each injection consisted of a 2.0% solution of WGA-HRP combined with a 10.0% solution of HRP dissolved in dH_2_O. The injection volume ranged from 0.01 μl to 0.05 μl. Two additional animals just received the PPRF injection of WGA-HRP.

Within 48 h of the PPRF injection, animals were perfused and their brains were sectioned at 100 μm, as described above. Tissue sections were reacted first to demonstrate the HRP reaction product (Olucha et al., 1985; Perkins et al., 2009). The sections were rinsed with 0.1 M, pH 6.0 PB. This was followed by incubation in a chromagen for 20 min: 5% tetramethylbenzidine (TMB; Free Base) in a solution containing 0.025% ethanol and 0.25% ammonium molybdate in the 0.1, pH 6.0 PB. Next, the sections were reacted by the addition of 0.3% H_2_0_2_ solution (0.011% final concentration) at room temperature for 1 h. The sections continued to react overnight at 4°C, with gentle agitation. They were then transferred to a stabilizer solution of 5.0% ammonium molybdate in 0.1 M, pH 6.0 PB, for 15 min, followed by multiple buffer rinses. Sections from animals that had only received the PPRF injection were mounted, counterstained and coverslipped at this point. Sections from the dual injection cases were then incubated in a solution of DAB in 0.1 M, pH 7.2 PB and reacted with addition of 0.3% H_2_O_2_ (0.011% final concentration) to stabilize the TMB reaction product. The tissue was then rinsed in buffer and reacted to reveal the BDA following the procedures outlined above.

### Analysis

For light microscopy, the distribution and morphology of anterogradely labeled terminals and retrogradely labeled neurons were charted using Olympus BH-2 or Nikon Eclipse 80i microscopes equipped with drawing tubes. Selected areas containing labeled terminals and/or neurons were digitally photographed with a Nikon Eclipse E600 photomicroscope equipped with a Nikon Digital DXM1200F color camera and Nikon Elements analysis software. In some cases, images from multiple focal planes were digitally combined using the Nikon Elements *Z*-axis program. The digitized images were adjusted in Adobe Photoshop to appear as close as possible to the visualized image.

For electron microscopic (EM) analysis, ultrathin sections were examined, and labeled profiles were photographed with a transmission electron microscope (Zeiss LEO 906). EM photographs of terminals were generally taken at magnifications of 21,560×. For characterizing labeling after postembedding immunohistochemistry for GABA, the number of gold particles in a 0.25 μm^2^ square sampled from 3 or more regions over axon myelin sheaths, per grid, was counted to provide a background particle density for use as a baseline. We classified the terminals into GABA-positive (GABA^+^, ≥3× baseline), intermediate (>baseline and <3× baseline) and GABA-negative (GABA^−^, ≤ baseline) categories. Somatic and dendritic profiles were classified into GABA^+^ (≥2 × baseline), intermediate (>baseline and <2× baseline) and GABA^−^ (≤ baseline).

## Results

### Anterograde Studies

**Figure 1:**
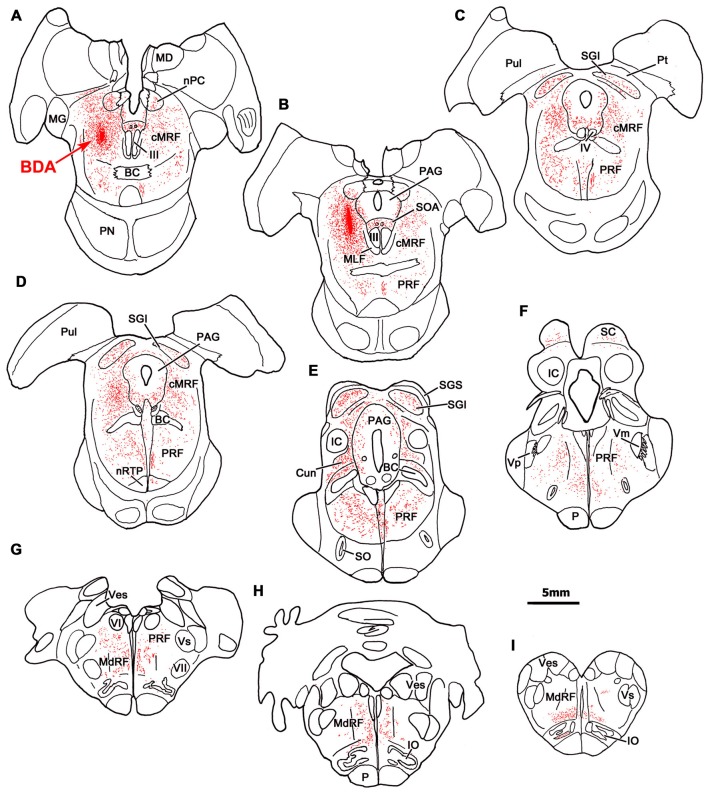
**Distribution of central mesencephalic reticular formation (cMRF) terminals labeled by biotinylated dextran amine (BDA).** The distribution of labeled terminals (stipple) and axons (lines) observed following a BDA injection into the cMRF **(A,B)** is charted on a rostral to caudal series of sections through the midbrain **(A–F)**, pons **(A–G)** and medulla **(G–I)**.

An example of a BDA injection in the cMRF is illustrated in Figure 1. This injection site lay in the left cMRF (Figures 1A,B) and it was largely confined to the center of the nucleus. A small amount of BDA was found within the needle track, which extended through the caudal pulvinar and part of the posterior pretectal nucleus (PPt). Within the midbrain, BDA labeled terminals (stipple) were found bilaterally, with an ipsilateral predominance, in the nucleus of posterior commissure (nPC; Figures 1A,B). A relatively intense terminal field was present in the lateral part of periaqueductal gray (PAG; Figures 1B–E) and the supraoculomotor area (SOA; Figures 1A,B), but few terminals were found in the oculomotor nucleus (III), as reported recently (Bohlen et al., 2016). A fairly dense terminal field was present in the contralateral cMRF (Figures 1A–D), which may underlie the presence of an inhibitory off direction in tonically active cMRF neurons (Waitzman et al., 1996). More caudally, an extensive terminal field was found in the SC (Figures 1C–F). Terminals were distributed to both sides, and were densest within intermediate gray layer (SGI), in agreement with previous reports (Zhou et al., 2008; Wang et al., 2010). Labeled fibers extended caudally from the cMRF injection site and terminated densely and bilaterally in the cuneiform nucleus (Cun; Figure 1E).

In the pons, labeled terminals were found throughout the PRF, including both its medially located magnocellular division and, to a lesser extent, in the laterally located parvocellular division. These terminals distributed to both sides, with an ipsilateral predominance (Figures 1B–G). On the ipsilateral side, numerous fibers coursed diagonally, dorsolateral to ventromedial in the rostral end of the PRF (Figures 1B–E), before taking up a medial location. On the contralateral side, labeled axons that had decussated beneath the rostral pole of III took up a position just off the midline, beneath the brachium conjunctivum (Figures 1A,B), and maintained this position, running just lateral to the raphe nuclei through the pons and into the medulla. They terminated extensively in the nucleus reticularis tegmenti pontis (nRTP; Figure 1D). Labeled terminals were densest in the rostral, ipsilateral PRF (Figures 1C,D). More caudally, the ipsilateral PRF terminals were concentrated medially, ventral and rostral to the abducens nucleus, where the horizontal gaze center is located (Figures 1E–G). However, scattered terminations were also present throughout the parvocellular regions found laterally in the PRF. On the contralateral side, there were fewer terminal arbors and they tended to be concentrated medially in the PPRF (Figures 1D–G). This trend becomes more evident as one precedes caudally. Numerous labeled terminals were present on the midline in the nucleus raphe interpositus (RIP), where the omnipause neurons lie (Figures 1C–E), as has been reported previously (Wang et al., 2013). Finally, BDA labeled terminals were present in the parabrachial nuclei (PB), mainly ipsilaterally (Figure 1E).

In the medulla, labeled cMRF terminals continued to target the reticular formation bilaterally (Figures 1G–I), but far more were found ipsilaterally, as previously described (Perkins et al., 2009). In the rostral medulla (Figure 1H), terminals were present dorsally in the medullary reticular formation (MdRF), where the IBN component of horizontal gaze center is located. The vast majority of MdRF terminals were located dorsal to the inferior olive (IO), particularly at more caudal levels (Figures 1G–I). Terminals were quite evident ipsilaterally, in the medial accessory nucleus of the IO (Figure 1I). Caudal to the area illustrated, the quantity of labeled terminals dropped off dramatically, so that only a small number of labeled terminals were present in the cervical spinal cord (for details, see Warren et al., 2008; Perkins et al., 2009).

**Figure 2:**
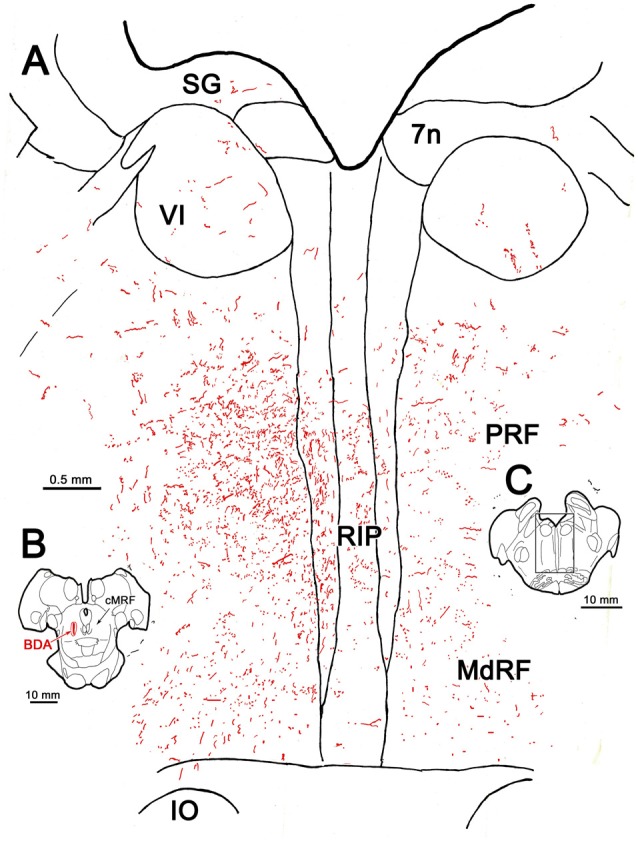
**BDA labeled reticuloreticular axons at the level of the abducens nucleus.** The pattern of reticuloreticular axons (lines) and terminals (stipple) is illustrated for the region shown by a box in **(C)** at higher magnification in **(A)**. The center of the BDA injection within the cMRF that produced the labeling is shown in **(B)**. Note the ipsilateral predominance of the terminal labeling in the pontine reticular formation (PRF) and medullary reticular formation (MdRF).

A more detailed example of the pattern of terminal labeling at the level of the abducens nucleus is presented in Figure 2. In this case, the BDA injection was slightly larger than the case shown in Figure 1, but was still largely confined to the cMRF (Figure 2B). A few axonal arbors were located within the abducens nuclei (VI) on both sides (Figure 2A). RIP was filled with many small labeled boutons that were often organized in clusters. Within the PRF, the ipsilateral labeling was distinctly denser, and most of the terminal labeling was distributed medially (Figure 2A). On both sides, thicker, dorsoventrally oriented axons were found just lateral to the raphe nucleus. These were relatively short, indicating their rostrocaudal course through a frontal section. The presence of these axons suggests that the descending projections of cMRF travel in a position similar to that of the predorsal bundle, which contains crossed tectobulbospinal axons. Thinner axons extended mediolaterally, presumably branches of these parent axons. Numerous fine axons with small boutons filled the neuropil ventral to VI, in the area of the PPRF. Only a few labeled axons and terminals were observed more laterally, in the parvocellular reticular formation. More ventrally in the section, labeled axons were found within the MdRF. These tended to run obliquely, dorsal to the IO.

**Figure 3:**
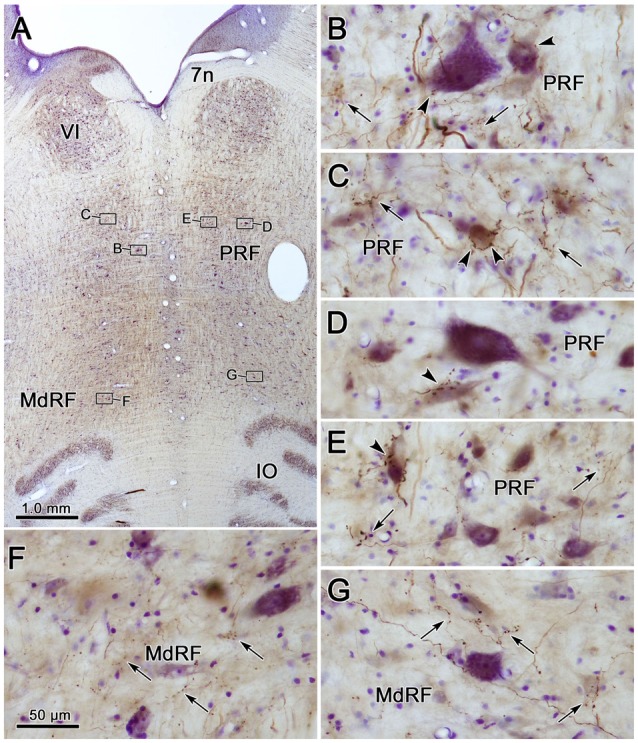
**Morphology of reticuloreticular axons labeled by BDA. (A)** Low magnification photomicrograph of a section through the abducens nucleus showing the location of the higher magnification plates in this figure (labeled boxes). Some of the BDA labeled axons displayed close associations with counterstained somata (arrowheads), while others terminated in the neuropil (arrows). The images were taken from the ipsilateral PRF **(B,C)**, contralateral PRF **(D,E)**, ipsilateral MdRF **(F)** and contralateral MdRF **(G)**. The case shown is the same as illustrated in Figure 2. (Number of 1.0 μm *Z* axis planes merged: **B,C,E** = 3, **D,F** = 1, **G** = 4).

Examples of the terminal patterns produced by BDA injection of the cMRF shown in Figure 2B are found in Figure 3. A section through VI is shown for reference (Figure 3A). Within the ipsilateral PRF, small, BDA labeled boutons formed close associations (arrowheads) with both the larger (Figure 3B) and more commonly the smaller (Figures 3B,C) cresyl violet stained somata, but most terminated in the neuropil. Thick labeled axons coursed through the neuropil and finer axons formed terminal arbors (arrows; Figures 3B,C). A similar pattern was observed in the contralateral PRF. There, close associations (arrowheads) between the small, BDA labeled boutons and cresyl violet stained somata were evident (Figures 3D,E), with most being related to the smaller cells. The axosomatic contacts were more evident contralaterally, but the main target of the terminal arbors (arrows) was the neuropil. A fine network of labeled axons was found within the MdRF, just dorsal to the IO (Figures 3F,G). Almost all the terminal boutons were located in the neuropil, and were not associated with counterstained somata. Fewer terminals were present on the contralateral (Figure 3G) than the ipsilateral side (Figure 3F).

**Figure 4:**
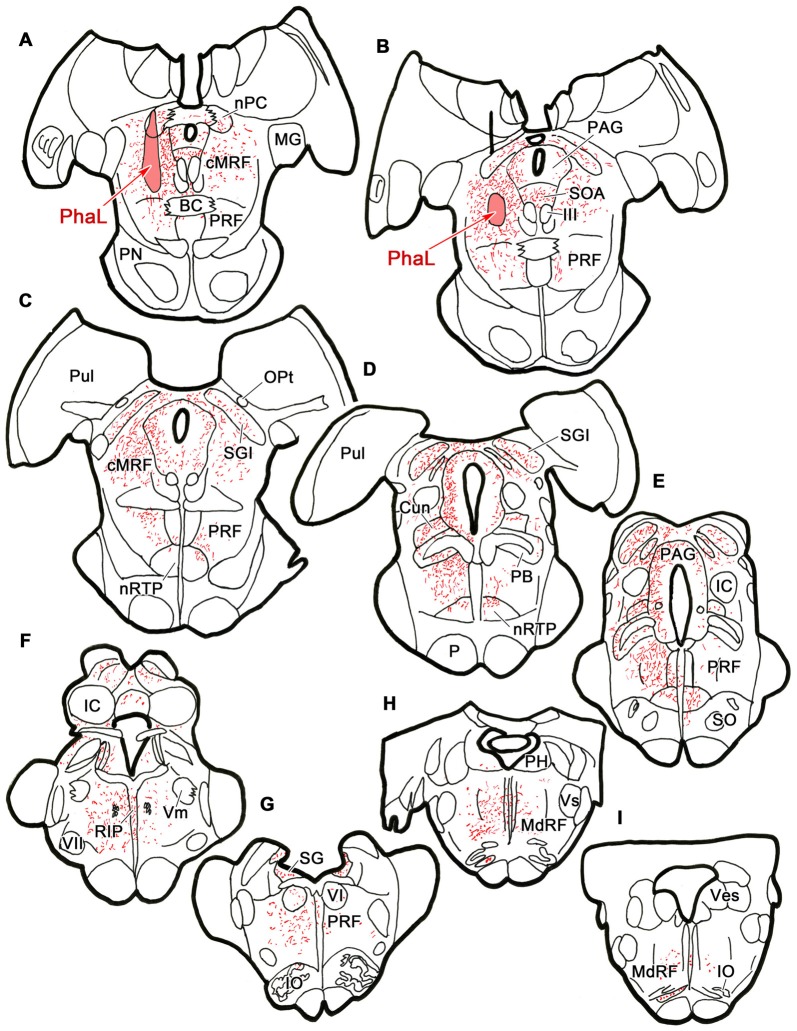
**Distribution of cMRF terminals labeled by *Phaseolus vulgaris* leucoagglutinin (PhaL).** The distribution of labeled terminals (stipple) and axons (lines) observed following a PhaL injection into the cMRF **(A,B)** is charted on a rostral to caudal series of sections through the midbrain **(A–F)**, pons **(A–G)** and medulla **(H–I)**.

To ensure that the pattern of label seen with the BDA injections was not due to fiber-of-passage uptake, we also performed PhaL injections, as this tracer shows little fiber-of-passage uptake (Gerfen and Sawchenko, 1984). In the illustrated case, the PhaL injection into the left cMRF was centered within the region, and included much of its dorsoventral extent (Figures 4A,B). It extended into the pretectum. In the rostral midbrain, PhaL labeled cMRF axon terminals (stipple) were found bilaterally, with an ipsilateral predominance. Their pattern of termination (Figures 4A–G) was very similar to that seen with the BDA injections (Figure 1). In the pons, large numbers of terminals were found in the PRF (Figures 4B–G). Far more axons and terminal boutons were located on the ipsilateral side, and the density of termination decreased, and became more medially concentrated at more caudal levels. Notably less evident was the labeled fiber track found in a paramedian position on the contralateral side following the BDA injection. This suggests this track mainly represented predorsal bundle axons labeled via fiber-of-passage uptake of BDA. Terminals were present throughout RIP (Figures 4F–H). Fewer labeled terminals were observed within the medulla (Figures 4H,I) than in the pons, but the ipsilateral predominance was still evident.

**Figure 5:**
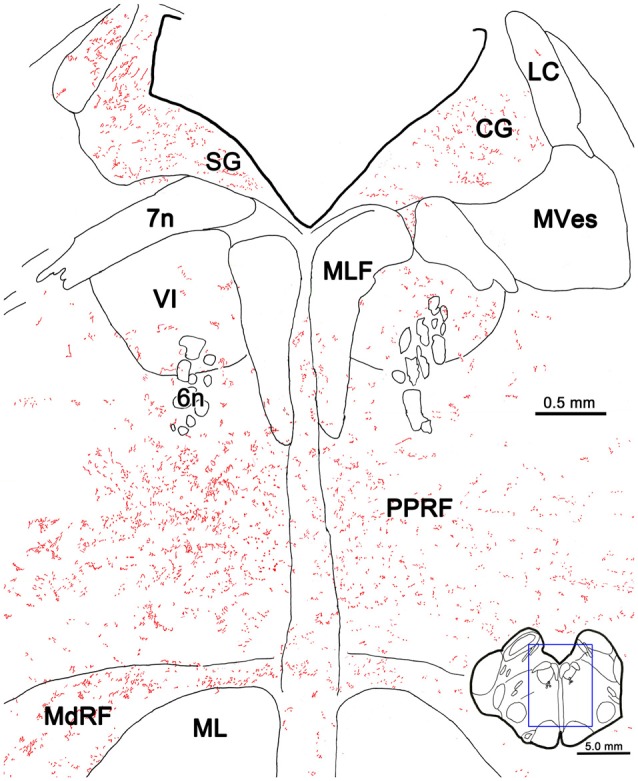
**PhaL labeled reticuloreticular axons at the level of the abducens nucleus.** The pattern of reticuloreticular axons (lines) and terminals (stipple) is illustrated for the region shown by a box in the inset. The injection site is illustrated in Figure 4. Note the ipsilateral predominance of the terminal labeling in the PRF and MdRF.

The pattern of axonal labeling following this PhaL injection is shown in greater detail in an illustration of a section at the level of the abducens nucleus (Figure 5). Note the relatively extensive labeling in the supragenual region (SG) above the facial nerve. There were a few terminal arbors within both abducens nuclei, but they were scattered. The main terminal field was in the ipsilateral PRF. This field was densest medially, within the PPRF. Labeled terminal arbors were also present contralaterally, but they were considerably fewer in number. The labeling within the MdRF was consistent with the BDA cases (Figure 2).

**Figure 6:**
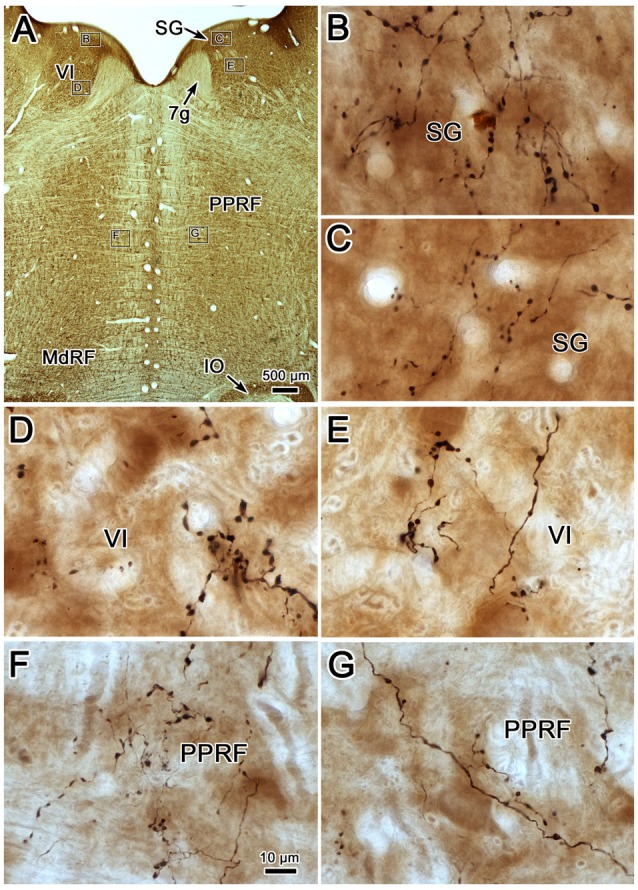
**Morphology of reticuloreticular axons labeled by PhaL. (A)** Low magnification photomicrograph of a section through the abducens nucleus showing the location of the higher magnification plates (labeled boxes) in this uncounterstained section. The images were taken from the ipsilateral and contralateral supragenual region (SG; **B,C**, respectively), ipsilateral and contralateral abducens nucleus (VI) (**D,E**, respectively), and ipsilateral and contralateral PRF (**F,G**, respectively). Note the larger number of boutons ipsilaterally in the PRF. The case shown is the same as illustrated in Figures 4, 5. (Number of 1.0 μm *Z* axis planes merged: **B,E,G** = 10, **C,F** = 13, **D** = 4).

Photomicrographs showing examples of PhaL labeled terminal arbors from the level of the caudal abducens nucleus (Figure 6A) are presented in Figures 6B–G. Thin, PhaL labeled axons could be observed with occasional branch points and terminal arbors within this region. The numerous *en passant* and terminal boutons varied in diameter. The density of the labeling was always heavier on the ipsilateral side (Figures 6B,D,F) than the contralateral side (Figures 6C,E,G). In the abducens nucleus, the boutons were clustered near individual cells, but most of the cells in the nucleus did not show adjacent terminals (Figures 6D,E). However, a dense network of terminals was observed dorsally, in the SG (Figures 6B,C). (Note that this is a complex region, that includes a number of supragenual nuclei (Büttner-Ennever et al., 1989; Biazoli et al., 2006; McCrea and Horn, 2006), but we will not examine this in detail here.) As can be seen in the PPRF samples (Figures 6F,G), the ipsilateral terminals seem more widespread in the neuropil, while associations with somata are more common contralaterally.

### Terminal Ultrastructure

**Figure 7:**
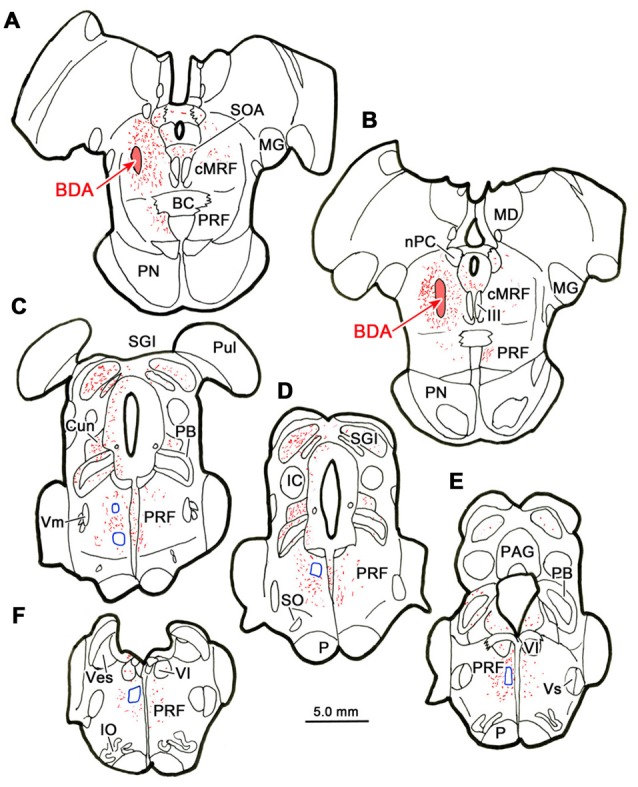
**Location of electron microscopic (EM) samples from the PRF.** The distribution of labeled terminals (stipple) and axons (lines) observed following a small BDA injection into the cMRF **(A,B)** is charted on a rostral to caudal series of sections through the midbrain **(A–E)** and pons **(A–F)**. The location of areas sampled for electron microscopy is indicated by blue boxes.

**Figure 8:**
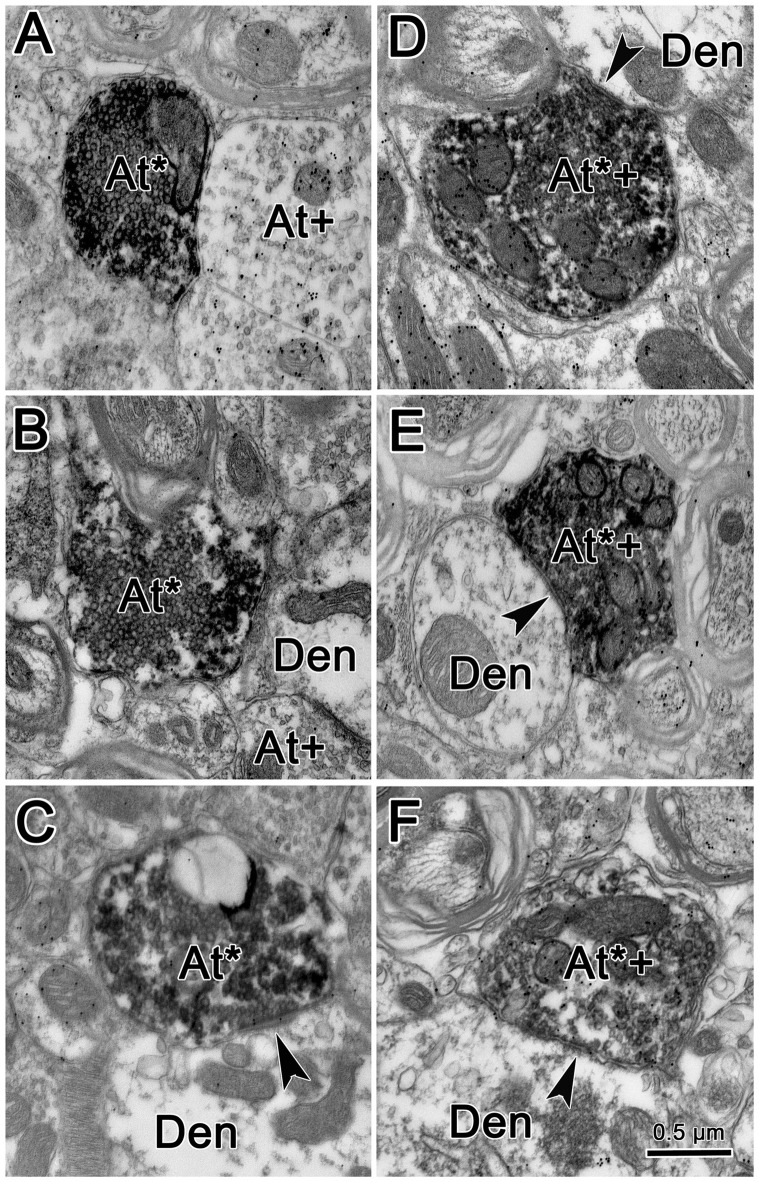
**Ultrastructure of reticuloreticular axon terminals.** Axon terminals that were labeled with BDA (At*) following the injection of the cMRF shown in Figure 7 were electron dense. Most of these terminals contacted (arrowheads) small dendrites (Den). These labeled terminals were heterogeneous: some were packed with clear spherical vesicles **(A–C)** and displayed asymmetric synaptic densities **(C)**. Others contained pleomorphic vesicles and made symmetric contacts **(D–F)**. Postembedding immunohistochemistry for gamma-aminobutyric acid (GABA) labeled a portion of the BDA labeled axon terminals (At^*+^), as well as some terminals not labeled with BDA (At^+^). The BDA labeled terminals that were overlain with numerous gold particles indicating they were GABAergic contained pleomorphic vesicles and made symmetric synaptic contacts **(D–F)**.

Figure 7 shows an example from a BDA case with a pair of small injections located laterally in the cMRF (Figures 7A,B). While the pattern of terminal label was similar to that described above, the number of labeled terminals was far smaller. The small windows in this figure indicate the areas where EM samples were taken. We concentrated our analysis on ipsilateral samples, where we expected to find solely inhibitory contacts. BDA labeled terminals (At*) were observed in material from the ipsilateral PPRF (Figure 8). Due to the BDA reaction product, they had greater electron density than profiles in the surrounding neuropil. Their morphological characteristics were similar to those we have described in the SC and RIP following cMRF injections (Wang et al., 2010, 2013). They were roughly round or oval in shape, and ranged in size between 0.40 μm and 3.60 μm along their long axis. These profiles were densely packed with small, clear vesicles that were either pleomorphic (Figures 8D–F) or spherical (Figures 8A–C) in shape. No dense core vesicles were seen in these terminals. The labeled terminals mostly contacted dendritic profiles. Both symmetric (Figures 8D–F) and slightly asymmetric (Figure 8C) synaptic densities were observed between the labeled terminals and dendritic profiles.

For brevity, the characteristics of the BDA labeled terminals are demonstrated here from examples observed following GABA postembedding immunohistochemistry. BDA labeled profiles that were judged to be either GABA-positive (GABA^+^) or GABA-negative (GABA^−^) were both found in the ipsilateral PPRF. Figures 8D–F shows examples of these BDA labeled, GABA^+^ terminals (At^*+^). They were overlain by numerous gold particles, indicating their GABA-positive nature. Most of them presented with pleomorphic vesicles. Symmetric synapses were observed between these terminals and GABA^−^ dendritic profiles (arrowheads). BDA labeled, GABA^−^ terminals are shown in Figures 8A–C. These BDA labeled terminals were overlain by very few, if any, gold particles (At*), compared to unlabeled, GABA^+^ terminals in the area (At^+^). Often, these terminals were nearly filled with densely packed, slightly larger, round, clear vesicles. These vesicle characteristics of homogeneity and high density were present in more than 90% of BDA labeled, GABA^−^ terminals throughout our EM samples. In Figure 8C, an asymmetric synapse (arrowhead) is shown between a BDA-labeled, GABA^−^ terminal (At*) and a GABA^−^ dendritic profile (Den).

We quantified the sample of terminals we observed. Among 64 terminals we observed in BDA/GABA double labeled material in the ipsilateral PPRF, 53.13% (34) were identified as GABA^−^, 35.93% (23) proved to be GABA^+^, while 10.94% (7) fell into the intermediate (undefined) category. All the postsynaptic elements contacted by these BDA labeled profiles were GABA-negative. Among all the BDA labeled terminals observed in both BDA labeled material and BDA/GABA labeled material, the vast majority contacted dendritic profiles.

### Retrograde Study

**Figure 9:**
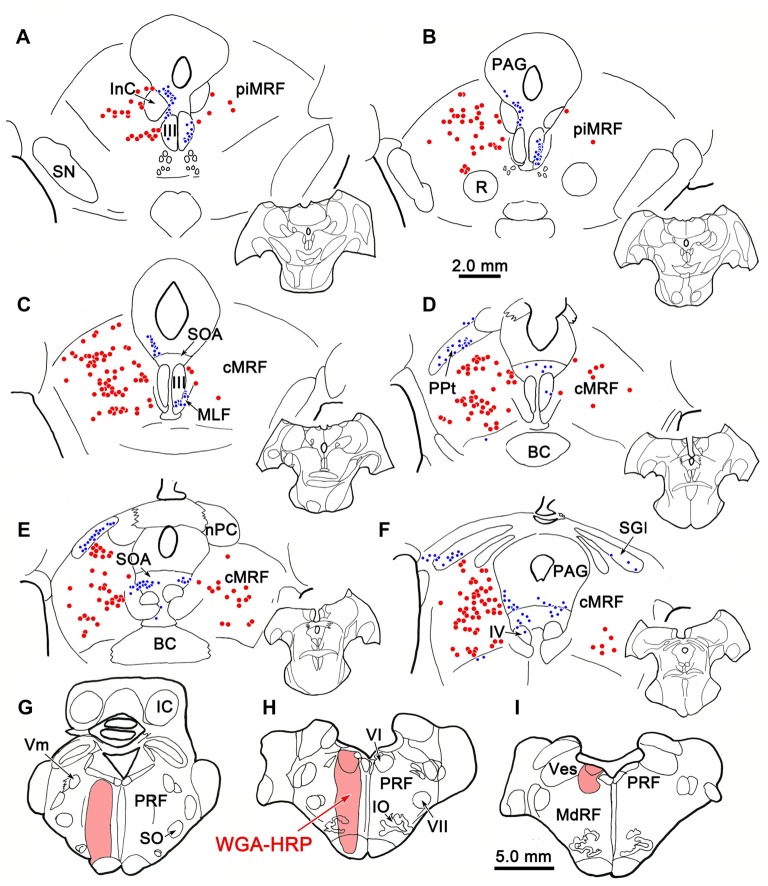
**Distribution of midbrain reticuloreticular neurons charted on a rostral to caudal series of sections.** An injection of wheat germ agglutinin conjugated to horseradish peroxidase (WGA-HRP) centered in the PRF **(G–I)** labeled reticuloreticular neurons (red dots) in the cMRF **(C–F)** and the piMRF **(A,B)**, as well as other midbrain cells (blue dots). Note the primarily ipsilateral distribution. Adjacent insets show the level of the selected sections.

Figure 9 shows the location of a WGA-HRP injection placed in the PRF and the distribution of the resultant retrogradely labeled neurons. The injection site involved the core of the PRF at the level of the abducens nucleus, and spread rostral to this level (Figures 9G–I). It included the abducens nucleus dorsally, and at its ventral end, spread slightly into the medullary reticular formation and IO. WGA-HRP labeled cells were observed within the cMRF (red dots) bilaterally (Figures 9C–F). However, the clear majority of the labeled neurons were located ipsilaterally. The dorsoventral spread of labeled cells extended throughout the entire MRF, so it included areas dorsal and particularly ventral to the region connected to the SC that has previously been defined as the cMRF (Chen and May, 2000). Labeled cells were also present with an ipsilateral predominance adjacent to the interstitial nucleus of Cajal (InC), in the piMRF (Figures 9A,B). Figure 9 also shows retrogradely labeled cells in other midbrain structures (blue dots). They were located in the PAG, the SOA and within III. A number of labeled cells were present in the ipsilateral PPt (Figure 9D). In a second case (not illustrated), the injection site was located just off the midline, and it produced more labeled cells in the contralateral than ipsilateral cMRF; but in this case, the injection site extended slightly across the midline.

### Dual Tracer Study

**Figure 10:**
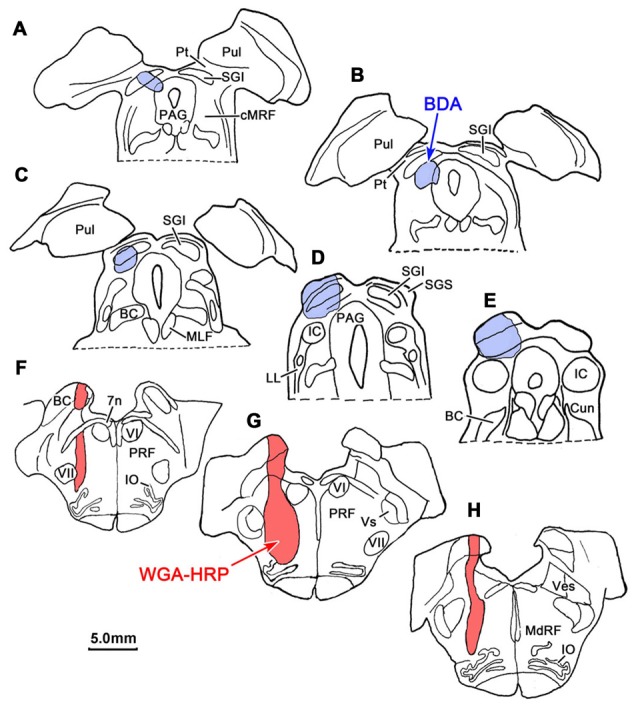
**Injection sites for a dual tracer case indicated on a rostral to caudal series of sections.** BDA was injected into the superior colliculus (SC; Blue area in **A–E**) and WGA-HRP was injected into the lateral PRF (Red area in **F–H**).

**Figure 11:**
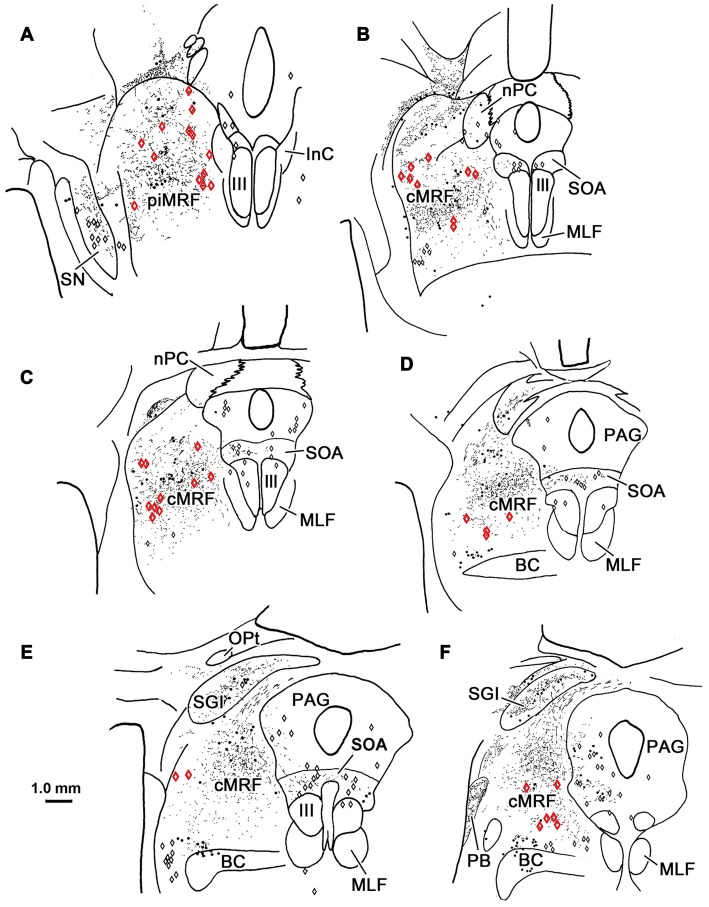
**Overlapping distribution of tectoreticular terminals and reticuloreticular neurons.** The distribution of tectal terminals (stipple) labeled by BDA and reticuloreticular neurons (red diamonds) labeled by WGA-HRP that resulted from the injections illustrated in Figure 10 are shown for the ipsilateral midbrain reticular formation (MRF). Note the overlap in their distributions within the cMRF **(B–F)** and the piMRF **(A)**. The reticuloreticular cells were scattered amongst BDA labeled reticulotectal neurons (black dots) in the cMRF. Other WGA-HRP labeled cells are indicated by black diamonds.

BDA labeled tectoreticular terminals together with WGA-HRP labeled reticuloreticular neurons were identified within the ipsilateral cMRF following combined injections of BDA into the left SC and WGA-HRP into the left PRF in three monkeys. In the illustrated case, the WGA-HRP injection was centered in the PRF (Figures 10F–H). The BDA injected in the left SC involved portions of all the collicular layers, with the largest concentration of tracer centered in SGI (Figures 10A–E). At the rostral end of the SC, the tracer spread to include a small portion of the dorsolateral PAG (Figures 10A,B). The resultant distribution patterns of the labeled elements are plotted in Figure 11. The BDA labeled tectoreticular terminals (stipple) were distributed throughout the rostrocaudal extent of the cMRF. In addition, reticulotectal cells, which were retrogradely labeled from the BDA injection in the SC (black dots), were distributed along a mediolateral band within this terminal field, defining the core of the cMRF (Chen and May, 2000). The injection of WGA-HRP into the PRF retrogradely labeled numerous reticuloreticular neurons (red diamonds). These neurons were observed throughout the rostrocaudal and mediolateral extent of cMRF (Figures 11B–F), with a heavier concentration of labeled cells at more rostral levels, including the piMRF (Figure 11A). The distribution of the labeled reticuloreticular cells exhibited an obvious overlap with the distribution of BDA labeled tectoreticular terminals. Other labeled neurons from the WGA-HRP injection (small black diamonds) were also observed in the PAG, nPC, III and SOA (Figures 11A–F). A few WGA-HRP labeled neurons were also seen in the contralateral cMRF (not illustrated).

**Figure 12:**
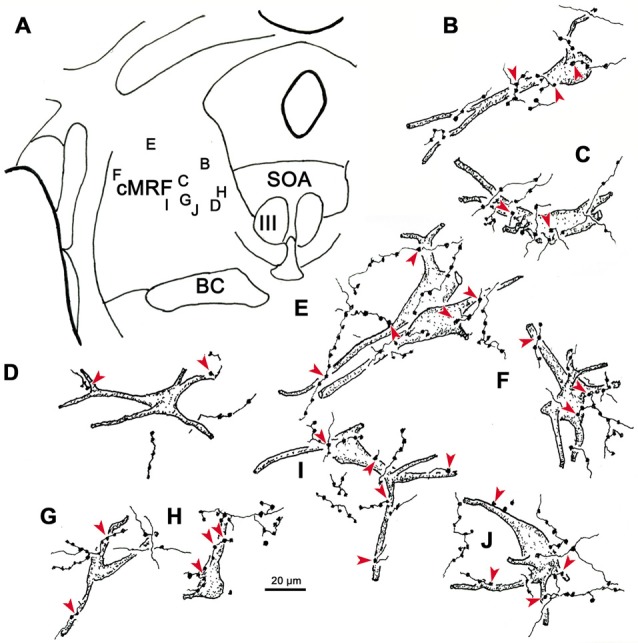
**The relationship between tectal inputs and reticuloreticular neurons in the cMRF.** Drawings reveal the presence of close associations (arrowheads) between BDA labeled tectoreticular terminals and reticuloreticular cells **(B–J)** labeled from a PRF injection of WGA-HRP. The location of the example cells within the cMRF is shown in **(A)**. While a number of close associations are present, the labeled axons rarely **(E,H)** follow the contours of the cell, and the number of contacts varied.

Examples of WGA-HRP labeled cells that were associated with BDA labeled terminals are illustrated in Figure 12. Their distribution in the cMRF is demonstrated in Figure 12A. These reticuloreticular neurons are multipolar neurons that possess three or more primary dendrites extending from their somata. Most were on the small side (10–20 μm for long axis). The BDA labeled terminals arbors consisted of thin fibers interrupted at numerous points by varicosities (boutons) of various sizes. These varicosities were seen in close association (arrowheads) with the somata (Cells B, C, E, F, H, I), as well as the proximal dendrites (Cells B–J), of the labeled reticuloreticular neurons. The close associations between the labeled boutons and neurons suggest synaptic contact, although some of the boutons sat above or below the labeled cell, and so were clearly in contact with other elements. It should be noted that some cells received only a few contacts (Cell D) and some received none at all (not illustrated).

**Figure 13:**
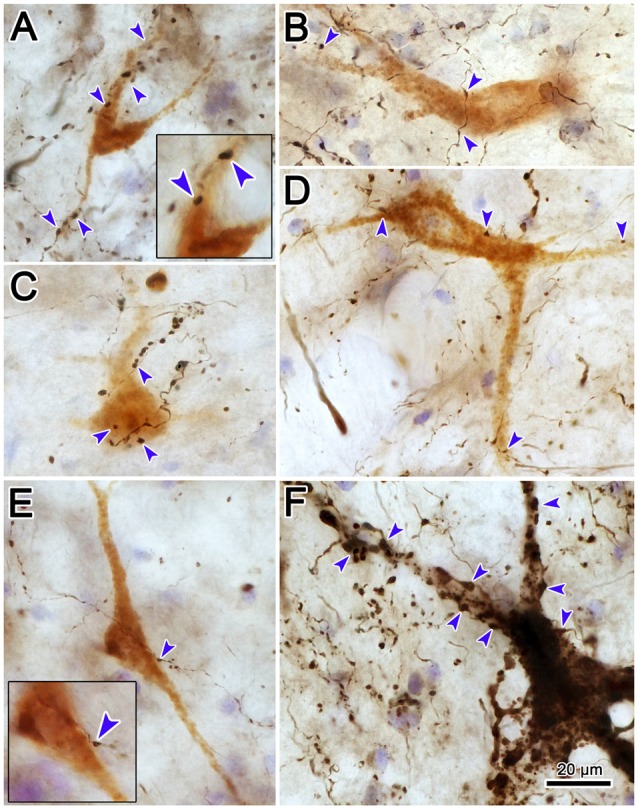
**Pattern of contacts between tectoreticular terminals and cMRF cells.** Photomicrographs show examples of close associations (arrowheads) between brown, retrogradely labeled reticuloreticular neurons and black, anterogradely labeled tectoreticular terminals **(A–E)**. Individual axons display one or two boutons associated with the cell in most **(B,D,E)**, but not all **(A,C)**, cases. In contrast, black, BDA labeled tectoreticular terminals line up along the dendrites of the black, BDA labeled reticulotectal neurons in the same case **(F)**.

High magnification photomicrographs (Figure 13) further demonstrate the relationship between the brown, WGA-HRP labeled reticuloreticular neurons and the black, BDA labeled tectoreticular axon terminals. The terminal boutons appear as beads on a string in the neuropil. These varicosities display close associations (arrowheads) with both the somata and dendrites of labeled reticuloreticular cells, although most are associated with unlabeled elements in the neuropil (Figures 13A–E). For comparison, Figure 13F shows an example of anterogradely labeled terminal boutons in close association with a reticulotectal neuron in cMRF that was retrogradely labeled from the BDA injection in the SC. The reticulotectal neurons were often larger than the reticuloreticular neurons. Furthermore, they were heavily invested with tectoreticular terminals that ran along their dendrites. Note that due to their color, the two classes of retrogradely labeled neuron could be easily discriminated.

## Discussion

The results of this study demonstrated that the descending reticuloreticular projections of the cMRF in *M. fascicularis* monkeys are concentrated in the PRF, where they display an ipsilateral predominance. The terminal field is densest in a paramedian position over the horizontal gaze center. By using GABA postembedding immunohistochemistry, it was revealed that cMRF sends both GABA^+^ and GABA^−^ ipsilateral projections to the region containing presaccadic, medium-lead burst neurons. These terminal types differ in their ultrastructure. The majority are GABA^−^ terminals that contain densely packed, spherical vesicles and make asymmetric contacts suggestive of excitatory input, while the minority are GABA^+^ terminals that have more dispersed, pleomorphic vesicles, and make symmetric contacts suggestive of inhibitory input. In the cMRF, the reticuloreticular cells that provide this output to the PPRF are relatively smaller, compared to reticulotectal cells. Close associations between BDA labeled tectoreticular terminals and some of these reticuloreticular cells suggest direct synaptic input. Thus, they are in a position to provide a conduit whereby tectal signals can gain access to the pontine gaze centers.

**Figure 14:**
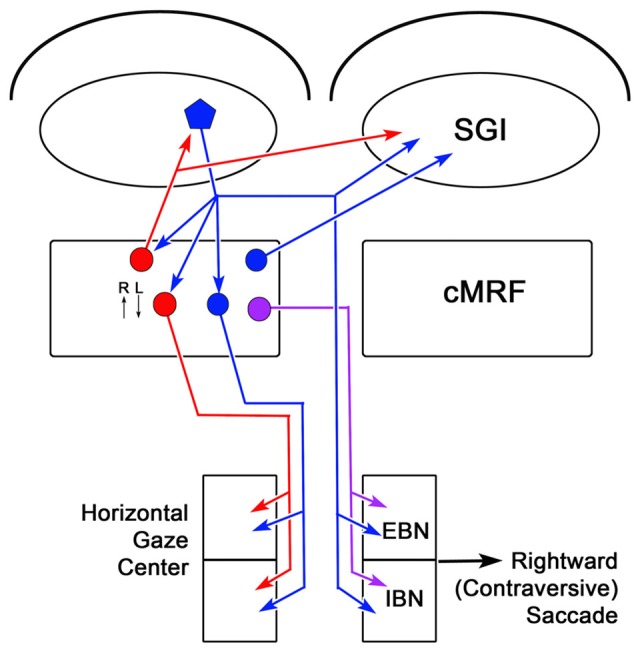
**Circuit diagram showing the cMRF as a conduit for collicular influence on lower brainstem centers.** Connections from the left side of the midbrain that would produce rightward (contraversive) saccades are shown. Neurons located in the cMRF receive direct input from the SGI of the SC and forward a signal to the horizontal gaze center, the brainstem center that controls horizontal saccadic eye movements. The horizontal gaze center contains both excitatory burst neurons (EBNs) and inhibitory burst neurons (IBNs) that are premotor cells projecting to the ipsilateral and contralateral abducens nucleus, respectively. Axons of collicular neurons (blue pentagon) that travel via the predorsal bundle, provide collaterals to the ipsilateral cMRF before crossing and terminating in the horizontal gaze center. The cMRF contains both inhibitory (red circle) and excitatory (blue circle) cells that project to the ipsilateral horizontal gaze center. In addition, it contains a population of cells (purple circle) that project to the contralateral horizontal gaze center. The cMRF also provides feedback to the SC, which is purely inhibitory (red circle), ipsilaterally, and both inhibitory and excitatory (red and blue circles, respectively), contralaterally. The activity of cMRF neurons for rightward (R) and leftward (L) saccades is indicated by small arrows.

The circuits that have been demonstrated by the present results and our previous work (Chen and May, 2000; Zhou et al., 2008; Wang et al., 2010) are illustrated in Figure 14. The SC projects to burst neurons in the contralateral horizontal gaze center to direct contraversive saccades. It also provides collaterals to the ipsilateral cMRF where neurons increase their firing for contraversive saccades and decrease their firing for ipsiversive saccades. The cMRF provides bilateral feedback to the SC. In addition, we have shown that it provides a crossed projection to the horizontal gaze center that is presumably excitatory and initiates a contraversive saccade. Within the cMRF, the tectal inputs targets two populations of ipsilaterally projecting reticuloreticular neurons. The inhibitory ipsilateral projection presumably silences the ipsilateral burst neurons during a contraversive saccade. The possible roles of the excitatory ipsilateral projection will be discussed below.

### Technical Considerations

One advantage of this study is that it utilized two different tracers to reveal the cMRF’s efferents, and showed labeled terminals in the same target structures. While false positive labeling due to tracer spread into areas outside the cMRF, particularly the pretectum, is possible, cross-case analysis showed that injections that did not involve these regions still produced the same basic pattern of terminal labeling. Labeling of axons of passage could also produce false positive labeling, as crossed tectobulbar projections (predorsal bundle axons) travel through the medial aspect of the cMRF. This does appear to have occurred with the BDA injections, as iontophoretic injections of PhaL, which produce little fiber of passage uptake, did not label the contralateral, paramedian axons (predorsal bundle) in the pons. Nevertheless, contralateral terminal fields were still present with PhaL injections.

In a postembedding immunohistochemical study, it is always possible that false positive and false negative labeling may be present. However, the fact that the antibody label was generally associated with a specific ultrastructural pattern that is characteristic of inhibitory synapses, and did not overlay terminals whose ultrastructural characteristics are generally associated with excitatory synapses, strongly suggests that our conclusion that the ipsilateral reticuloreticular projection to the pons is not exclusively inhibitory is correct. We found that all the targets of these cMRF terminals were GABA^−^, which suggests that cMRF axons contact cells other than GABAergic interneurons. This is not surprising, as IBNs use glycine as a transmitter (Spencer et al., 1989). Therefore, the targets of cMRF terminals in PPRF are very likely to be presaccadic burst neurons. We cannot tell from our results whether these target cells are LLBNs, EBNs or IBNs.

In the dual tracer experiments, the WGA-HRP injections of the pons spread into the MdRF. However, the reticuloreticular cells that supply the MdRF are restricted to the medial cMRF (Perkins et al., 2009), and we did not see this cell distribution pattern in the present study. These retrograde results support the presence and the degree of laterality of the reticuloreticular projection from the cMRF observed anterogradely. The injections of the SC, were relatively discrete, and so were unlikely to have produced false positive terminal labeling. In these experiments, we observed close associations between anterogradely labeled tectoreticular terminals and retrogradely labeled reticuloreticular neurons in the cMRF at the light microscopic level. While these close associations suggest synaptic contact, this assertion remains to be confirmed by ultrastructural analysis. Unfortunately, the collicular injections were too large to indicate any topography with respect to cMRF projections, such as has been reported by other means (Cohen et al., 1985).

### Comparison to Previous Findings

Similar to the pattern of anterograde label in the present study, Edwards (1975) found ^3^H-leucine labeled terminals in nRTP and in the rostral and caudal portions of the PRF after injecting tritiated amino acids into the Cun in cats. His Cun included the region termed the cMRF here. However, the projection observed in cats was more evenly bilateral than observed in the present findings. A fluorescent retrograde tracer study of inputs to feline PRF has also shown a stronger contralateral projection than we have seen in the present study (Perkins et al., 2009). On the other hand, Cowie and Robinson (1994) reported a primarily ipsilateral cMRF distribution in monkeys after injecting the rostral medullary reticular formation, similar to the present findings. Thus, there appears to be a species difference in the degree of laterality of the cMRF projection. Although there are differences in the orienting movements of the cat and macaque (Guitton et al., 1990; Fuller, 1992), a specific explanation for this difference in laterality must await a better understanding of the role of the ipsilateral reticuloreticular projection of the cMRF. We also illustrated a sparse, bilateral projection to the abducens nucleus, which we previously described (Bohlen et al., 2016). This projection was not seen in the cat (Edwards, 1975), most likely for technical reasons. When Ugolini et al. (2006) injected the retrograde transneuronal tracer, rabies virus, into the lateral rectus muscle of monkeys, they also found transneuronally labeled neurons in the medial portion of caudal cMRF, supporting our finding that the cMRF projects directly to abducens motoneurons.

The results of these experiments may also speak to the question of the relative importance of the feed forward and feedback circuits in cMRF function. Comparison of the numbers of labeled reticulotectal, reticulopontine and reticulomedullary neurons in the cMRF show consistent differences (Chen and May, 2000; Warren et al., 2008; Perkins et al., 2009, 2014; Wang et al., 2010, 2013; Present Results). In cats, reticulotectal neurons outnumbered the cells with descending axons (Perkins et al., 2014) and the reticuloreticular projection seen here in monkeys is less intense than the cMRF’s projections to the ipsilateral SC (Figures 1, 4, 9; Zhou et al., 2008; Wang et al., 2010). In addition, cMRF terminals tended to have a distal location on the dendritic tree of PRF neurons. Thus, the feedback signal may be more robust than the feed forward signal, and perhaps more central to cMRF function. An additional difference was noted here. The number of tectal terminals contacting individual cMRF neurons varied with respect to the target of the neuron. Specifically, the reticulotectal neurons had a far greater number of tectoreticular terminal associations (Figure 13E; Chen and May, 2000), when compared to the reticuloreticular neurons (Figures 12, 13A–D). The extensive tectal input onto cMRF reticulotectal neurons suggests that their physiologic characteristics are largely controlled by inputs from predorsal bundle axons. This anatomical finding correlates with the physiological descriptions of these neurons given by Moschovakis et al. (1988a), who suggested there was little difference between the firing characteristics of these cMRF cells and tectal LLBNs. In contrast, the more limited input observed on cMRF reticuloreticular neuron suggests that while the collicular input influences these cells, it is not nearly so dominant an input as that targeting the feedback circuit.

### The Contralateral cMRF-PPRF Projection

The PPRF is a primary target of the descending predorsal bundle axons from the SC (cat: Cowie and Holstege, 1992; monkey: Harting, 1977; Basso and May, 2017). In the present study, we have demonstrated that the horizontal gaze center is also targeted by the cMRF in monkeys. The terminations are more prevalent rostrally, which is where EBNs are more common, compared to caudally, where IBNs are more common. Since the SC provides the major input to the cMRF via collaterals of the predorsal bundle axons (Harting, 1977; Grantyn and Grantyn, 1982; Moschovakis et al., 1988a), then it is likely that it targets reticuloreticular neurons in the cMRF. Thus, the presence of a crossed cMRF projection to the PPRF, as shown here, supports the existence of a crossed tectoreticuloreticular pathway that traverses the cMRF, as proposed by Waitzman et al. (1996). They suggested that this pathway might help produce the spatiotemporal transformation needed to change the topographic code of the SC into the firing rate code of medium lead burst neurons. Further studies demonstrated that the firing of some cMRF cells do appear to represent a partial transformation of the collicular signal (Cromer and Waitzman, 2006, 2007). On the other hand, the cMRF terminals we observed often had a relatively distal distribution on pontine reticular neurons, suggesting a modulatory, as opposed to a driving influence central to the spatiotemporal transformation. Indeed, the function of this trans-cMRF pathway from the SC to the PPRF is not entirely clear. Muscimol inactivation of the cMRF, which leaves the collicular projections to the PPRF intact, but eliminates the trans-cMRF pathways, leads to hypermetric contralateral horizontal saccades (Waitzman et al., 2000b). This is not necessarily what one would expect if the cMRF is a key factor in the spatiotemporal transformation and if this effect is due to loss of downstream projections of the cMRF. Of course, the hypermetric saccades might be due to loss of feedback projections to the SC, although stimulation of the cMRF in animals whose ipsilateral SC has been ablated still produces horizontal eye movements, indicating that pathways from the cMRF are capable of inducing eye movements without any contribution of cMRF-SC circuits (Cohen et al., 1986; Luque et al., 2006).

### The Ipsilateral cMRF-PPRF Projection

It is interesting that the cMRF projection in the monkey mirrors the tectal projection, with the cMRF terminal field being predominantly ipsilateral and the collicular terminal field being predominantly contralateral (Figure 14). There is evidence that pathways from the ipsilateral SC influence activity observed in horizontal gaze center neurons, specifically IBNs (Strassman et al., 1986b; Sugiuchi et al., 2005; Takahashi et al., 2005). However, the authors of these articles ascribe most of these effects to tectotectal interactions, not tectoreticular pathways. If the collicular and cMRF pathways work together in a push-pull manner while producing a contraversive saccade, then the ipsilateral cMRF projection should be inhibitory, as the left PPRF is generally silent for a rightward movement, while the right PPRF is active (Hepp and Henn, 1983; Strassman et al., 1986a). This hypothesis is based on the fact that electrical stimulation of the cMRF in the monkey produces contraversive horizontal saccades (Cohen et al., 1985). Furthermore, when Cromer and Waitzman (2007) recorded the presaccadic responses of cMRF neurons, they found neurons that fired in association with contraversive horizontal saccades. Our observation of a GABA^+^ component among the cMRF axons that terminate in the ipsilateral PPRF is consistent with this directional preference. Many of the neurons recorded by Waitzman et al. (1996) showed a fairly high tonic firing rate, which was silenced by ipsiversive eye movements. If these cells represent the ipsilaterally projecting GABAergic population observed here, they could inhibit the activity of burst neurons in the ipsilateral PPRF during contraversive eye movements and so help to eliminate activity in antagonist eye muscles. This GABAergic input would be turned off for ipsiversive eye movements when this side of the PPRF would be activated.

Not all the findings are consistent with this push-pull hypothesis. Specifically, the ipsilateral projection is heterogeneous (GABA^+^ and GABA^−^), instead of purely inhibitory, as would be expected if the sole function of this projection was to drive contraversive eye movements. While the presence of an excitatory ipsilateral cMRF-PPRF projection could help to explain why inactivation of the cMRF also affected ipsiversive saccades, making them hypometric (Waitzman et al., 2000a), it is not clear what the role of this excitatory ipsilateral pathway is.

One possibility is that cMRF saccadic burst neurons are not the actual target of this excitatory ipsilateral projection. Pathmanathan et al. (2006a,b) described “postsaccadic” cMRF neurons, characterized by firing after gaze shift onset or at the end of the gaze shift. In head-free animals, the firing of these postsaccadic cMRF neurons was found to be most closely associated with head movements. In fact, the cMRF has mainly ipsilateral projections to the medullary reticular formation and spinal cord that presumably affect head movements (Warren et al., 2008; Perkins et al., 2009). Perhaps the GABA^−^ cMRF terminals found in the PPRF in the present study actually contact the dendrites of reticulospinal neurons. If this is true, the excitatory ipsilateral reticuloreticular projections could be there to adjust the activity of reticulospinal neurons that act to brake the movement of the head or maintain head position (Corneil et al., 2001; Perkins et al., 2009).

Another possibility comes from consideration of the fact that the heterogeneous pattern of cMRF termination in the ipsilateral PPRF resembles the pattern of cMRF termination in the contralateral SC (Figure 14; Wang et al., 2010). Both structures have been presumed to be silent during the production of saccades in the off direction. Perhaps, the excitatory ipsilateral cMRF projection to the PPRF, like the crossed cMRF reticulotectal projection, may function in organizing complex saccadic behaviors. Mays and Sparks (1980) utilized two targets to trigger sequential saccades. When they arranged the target sequence to produce a leftward and then a rightward saccade, the activity corresponding to the rightward saccade was in the left SGI, even though the visual activity all occurred in the right superficial gray layer. If the movement of the activity into the other side of the SC is transmitted by the crossed excitatory reticulotectal projection of the cMRF, direct excitation to the ipsilateral PPRF by the cMRF may be assisting the downstream effects of the crossed tectoreticular projection during such saccade sequences.

A third possibility that must be considered in light of the dominant, excitatory projection to the ipsilateral PRF is the idea that the cMRF supports behaviors other than orienting saccades. The SC is known to also help direct avoidance movements through its ipsilateral descending projections (Ingle, 1983; Dean et al., 1986; Ellard and Goodale, 1986; Furigo et al., 2010; Comoli et al., 2012). Perhaps the cMRF also takes part in directing the eyes and head away from threats. In this case, the ipsilateral cMRF projections would parallel the ipsilateral descending collicular projections. In effect, they would provide a pathway for supporting the spatiotemporal transformation in an avoidance movement, instead of an orienting movement. Arguing against this idea is the fact that Comoli et al. (2012) saw relatively few terminals in the MRF following collicular injections into the region associated with avoidance in rats.

A final possibility is suggested by recent findings that indicate that abducens motoneurons do not always act in a manner that can be predicted by a purely antagonistic and purely conjugate model. Indeed, there is evidence that some extraocular motoneurons appear to display signals that are better related to the contralateral eye (Zhou and King, 1998; Van Horn and Cullen, 2009). The mixed ipsilateral projections found in this study might help to explain these signals. These investigators have also provided evidence that medium lead burst neuron activity is also not purely predicted by conjugate movement models. During disjunctive saccades between targets that lie at different distances from the observer, the activity of an individual burst neuron in the left PPRF can be best correlated to the action of either the left or right eye (Zhou and King, 1998; Van Horn et al., 2008). Similar eye-specific signals have been observed on saccade-related cells in the cMRF (Waitzman et al., 2010). We have recently reported projections to the SOA that terminate on neurons in the Edinger-Westphal nucleus and medial rectus motoneurons in the C-group in a pattern that is consistent with the idea that the cMRF plays a role in disjunctive saccades, where each eye is independently directed to compensate for target distance (Bohlen et al., 2016, 2017; May et al., 2016). Thus, the heterogeneous cMRF projection to the ipsilateral PPRF may be part of this same system that modulates burst neuron activity during disjunctive saccades. In this light, it would be worth investigating whether the crossed cMRF projection also displays both GABAergic and non-GABAergic components.

In summary, the cMRF provides both excitatory and inhibitory inputs to burst neurons in the pontine gaze centers. Compared to the SC, its descending projections to the contralateral PPRF are less robust, but its ipsilateral projections are much stronger. The heterogeneity of the ipsilateral projection and the distribution of tectoreticular inputs on reticuloreticular neurons suggest that saccadic signals are not just relayed through the SC-cMRF-PPRF pathway. Instead, cMRF may modify the activity of presaccadic burst neurons on both the ipsilateral and contralateral side in a complex pattern. Considering the fact that the cMRF exerts its influence via both GABA^+^ and GABA^−^ terminals and has both ipsilateral and contralateral components, it is likely that it springs from several different cMRF cell populations that display various target-specific patterns of gaze-related activity. These should be a target of further investigation if we are to have a better idea of the role of the cMRF in gaze.

## Author Contributions

PJM: study concept and design; critical revision of the manuscript. NW, LZ and EP: acquisition of data; drafting the manuscript. NW, LZ, EP, SW and PJM: analysis and interpretation of the data. PJM and SW: obtaining funding; study supervision. All authors had full and open access to the data in the study and take responsibility for the integrity of the data and the accuracy of the data analysis.

## Funding

This research was supported by National Institutes of Health Grant: EY01426 to PJM and SW.

## Conflict of Interest Statement

The authors declare that the research was conducted in the absence of any commercial or financial relationships that could be construed as a potential conflict of interest.

## Abbreviations

6n: abducens nerve
7n: facial nerve
III: oculomotor nucleus
IV: trochlear nucleus
VI: abducens nucleus
VII: facial nucleus
At: axon terminal
BC: brachium conjuntivum
BDA: biotinylated dextran amine
CG: central gray
cMRF: central MRF
Cun: cuneiform nucleus
Den: dendrite
GABA: gamma amino butyric acid
HRP: horseradish peroxidase
IC: inferior colliculus
InC: interstitial nucleus of Cajal
IO: inferior olive
LC: locus ceruleus
LL: lateral lemniscus
MD: medial dorsal nucleus
MdRF: medullary reticular formation
MG: medial geniculate nucleus
ML: medial lemniscus
MLF: medial longitudinal fasciculus
MVes: medial vestibular nucleus
MRF: mesencephalic reticular formation
nPC: nucleus of the posterior commissure
nRTP: nucleus reticularis tegmenti pontis
OPt: olivary pretectal nucleus
P: pyramid
PAG: periaqueductal gray
PB: parabrachial nuclei
PH: nucleus prepositis hypoglossi
PhaL: *Phaseoulus vulgaris* leucoagglutinin
piMRF: peri-InC portion of the MRF
PN: pontine nuclei
PRF: pontine reticular formation
PPRF: paramedian pontine reticular formation
Pul: pulvinar
Pt: pretectum
PPt: posterior pretectal nucleus
R: red nucleus
RIP: nucleus raphe interpositus
SC: superior colliculus
SG: supragenual region
SGI: intermediate gray layer
SGS: superficial gray layer
SN: substantia nigra
SO: superior olive
SOA: supraoculomotor area
Ves: vestibular nucleus
Vm: trigeminal motor nucleus
Vp: principal trigeminal nucleus
Vs: spinal trigeminal nucleus
WGA-HRP: wheat germ agglutinin conjugated to horseradish peroxidase.
